# Prevention and control of mosquito-borne arboviral diseases: lessons learned from a school-based intervention in Brazil (Zikamob)

**DOI:** 10.1186/s12889-022-12554-w

**Published:** 2022-02-08

**Authors:** Silvana Santos, Roberta Smania-Marques, Victor Alves Albino, Izabelly Dutra Fernandes, Francisco Fernandes Abel Mangueira, Ruy Alberto Pisani Altafim, Ricardo Olinda, Matt Smith, John Traxler

**Affiliations:** 1 Public Health Programme, Universidade Estadual da Paraíba - Campus I - Bodocongó, Rua das Baraúnas, s/n - Prédio da Integração Acadêmica - sala 329, Campina Grande, 58490-500 Brazil; 2 Department of Biology, Universidade Estadual da Paraíba, Campina Grande, Brazil; 3 Secretary of Education, Science and Technology, State of Paraíba, João Pessoa, Brazil; 4 Department of Informatics, Federal University of Paraíba, João Pessoa, Brazil; 5 Department of Statistics, Universidade Estadual da Paraíba, Campina Grande, Brazil; 6 Education Observatory, University of Wolverhampton, Wolverhampton, UK

**Keywords:** Arbovirus, Health Education, Public Health, Behaviour Change Theories

## Abstract

**Background:**

Since the 1980s, when dengue was reintroduced in Brazil, outbreaks and epidemics caused by different arbovirus strains transmitted by vector mosquitoes such as *Aedes aegypti* have been an annual occurrence. The aim of this study was to evaluate the behavioural change of high school students and teachers who participated in an educational intervention for the prevention and vector control of arboviral diseases.

**Methods:**

In this school-based intervention, a self-reported questionnaire was used in a pre-post intervention methodology to assess environmental risk factors, sociodemographic variables and to measure attitudes and behaviours. In all, 883 high school students and teachers from the city of Campina Grande, in the state of Paraíba, northeastern Brazil, participated. The e-health intervention consisted of a competition between schools to comply with preventive actions via content production for social networks, and the monitoring was performed over a period of three months through the ZikaMob software developed by the researchers.

**Results:**

Out of the 883 survey participants, 690 were students ranging in age from 14 to 41 years, with an average of 17 ± 2 years; and 193 were teachers from 22 to 64 years old, averaging 38 ± 9 years. The analysis of the data shows that significant differences in most of the target behaviours were apparent after the intervention, with an increase of about 10% in the performance of inspection actions; a 7% greater separation of recyclables and a 40% increase in the screening of windows among students. Students showed lower fear of, and a lower self-perception of the risk of, acquiring arboviruses than teachers on average.

**Conclusions:**

ZikaMob is an innovative strategy with the potential to be replicated in any location that has an internet network and can involve an unlimited number of participants.

**Supplementary Information:**

The online version contains supplementary material available at 10.1186/s12889-022-12554-w.

## Background

Since the 1980s, when dengue was reintroduced in Brazil, outbreaks and epidemics caused by different arbovirus strains transmitted by vector mosquitoes such as *Aedes aegypti (A.e)* have been an annual occurrence [1]. Since 2010, it has been estimated that there have been over a million cases of dengue per year, leading to hundreds of deaths [2]. Since 2015, with the introduction of the Zika and Chikungunya viruses in the country, cases have been reported of children with congenital Zika syndrome or chronic sequelae [2]. The similarity of symptoms, cross-reactivity, co-circulation and overlap of infections by different arbovirus species and strains make the differential diagnosis of these diseases difficult [1]. The main strategy for reducing the prevalence of these diseases has been vector control with larvicide application in water tanks and spatial nebulization with insecticides widely used in the control of adult specimens [3], which has contributed to the selection of resistant insect strains [4]. The development of vaccines and alternative control methods using transgenic bacteria and mosquitoes has also been reported in the literature [5, 6].

The engagement of the population in preventive actions is one of the main objectives of vector control policies, given that most of the breeding sites are located in households and on vacant land and abandoned houses [7, 8]. This engagement has been stimulated and supported by mass media advertising, the use of breeding reporting applications (eg Zikazero) [9] and environmental education by home-visiting performed by health workers [7, 10]. Unfortunately, in urban centres in Brazil, these visits often do not occur because residents are not in their homes or they do not allow health workers to enter for inspection and guidance on vector control for fear of violence [11]. In this context, the implementation of interventions in schools using digital platforms for mobile devices can be a powerful tool for the promotion of engagement by the population in preventive actions for arboviruses, and this can be directed by entomological surveillance services.

The World Health Organization has recommended grounding educational interventions in behaviour change theories [12, 13], considering the growth of evidence for their effectiveness at individual, community and population levels [14, 15]. The Health Belief Model (HBM), for example, suggests that decision-making depends on the perception of susceptibility, disease severity, benefits and barriers associated with behaviour [12, 16]. Social Cognitive Theory (SCT) describes three main factors that affect a person's likelihood of changing health behaviour: self-efficacy, goals, and outcome expectations [12, 17]. Even small changes in human behaviours can have substantial effects on population health outcomes [13].

Building on these behaviour change theories, the aim of this study was to evaluate the behavioural change of high school students and teachers who participated in a virtual educational intervention. This is the third stage of the project, “Impact of mobile learning on prevention and management of complications caused by arbovirus (Zika, Dengue, Chikungunya) – ZikaMob,” funded by the British Council and the Government of Paraíba State, in Northeastern Brazil.

### Design and scenario

This is a school-based intervention in which a self-reported questionnaire was used before and after the intervention to assess environmental risk factors and sociodemographic variables, and to measure attitudes and behaviours. The theoretical and methodological foundations of the research, the software development process and the validation of the data collection instrument were described previously [18–20].

Campina Grande is the second largest city in the state of Paraíba, housing an estimated population of 409,731 in 2019, with 15,152 students and 1,732 teachers registered in high schools (IBGE). The city is in the semiarid northeastern region and experiences prolonged dry periods; for this reason, the state periodically establishes water rationing, ranging from two to four days a week, causing the population to use water tanks or buckets for water storage. From 2018, with the transposition of waters of the São Francisco River, there was a recharge of water in supply dams with a concomitant suspension of the water supply rotation. The city had high rates of arbovirus vector mosquito infestation, with an associated high risk for arbovirus outbreaks and epidemics [20].

### Intervention

The intervention consisted of a competition between high schools in order to fulfill the largest number of arbovirus prevention educational activities (“missions”). A virtual platform called *ZikaMob* was established to allow the follow-up of the execution of these “missions” and the inclusion of audio-visual content published on social networks. To participate in the project, students were registered on the platform through an invitation sent to their personal email and following their guardians’ signing of the consent form. Each participant had an individual password, could access the platform from any mobile device and were able to track activities virtually or via the Zikamob Facebook page.

The Zikamob platform is a virtual learning environment, like Moodle or Google Classroom, allowing the inclusion of didactic material, quizzes, questionnaires, and videos. Unlike existing platforms, on Zikamob participants could include the Facebook URLs of their videos to prove that they had completed the mission. By including their social media posts or answering the questionnaires on the Zikamob platform, the student received points. The sum of students' points was used to compose the total points for each school. The individual and school points were counted on the platform as well as likes, shares and interactions of their social media posts.

Every two weeks, participants were given a mission and had to produce a creative video to prove their accomplishment and post it on Facebook. In all, five video missions and two questionnaires were completed during a three-month period. The first mission was to make a video inviting people to follow the Zikamob Facebook page (https://www.facebook.com/zikamob.uepb.3) and to engage in the activities. The second mission was to learn how to inspect the home, identify mosquito-borne arbovirus outbreaks, and how to properly eliminate them. Students had to watch a report with a health worker that explained how to complete these procedures. The third mission was to learn how to sort recyclables and donate to waste pickers, using an application called CATAKI that lets users locate waste pickers in order to match the delivery of recyclable material. In Campina Grande, like most Brazilian cities, there is no selective collection service and the trash found in the streets, backyards and open areas serves as fertile breeding areas for mosquitoes. The fourth mission was to learn how to screen windows and drains to prevent mosquitoes from entering homes. The final mission asked participants to make clean-up efforts in their backyards or vacant lots near their homes.

The ZikaMob project's Facebook page allowed the publication of calendars, warnings, and mission guidance. In schools, each class selected the top three videos, and these received extra points. Teachers were responsible for choosing the top three videos from the school for each of the missions, which also received extra points. A video representing each school in each of the five missions was posted on the ZikaMob Facebook page for the public to vote on through the awarding of likes. The three videos with the most likes for each mission were nominated for awards, for which the winners received cell phones and other awards. The observation of people taking preventive action is one of the factors that can promote desired behaviour change, according to the Social Cognitive Theory [12].

The project was initiated after approval by the Research Ethics Committee of the State University of Paraíba (Protocol CAAE 67429517.5.0000.5187) and due consent of the participants’ parents or guardians was given through the signing of the informed consent form. The registration of teachers and students in the ZikaMob platform was carried out from April to June, and the intervention during the months of July to September 2019.

### Evaluation Instrument

The assessment of perceptions and behaviours before and after the intervention was performed by applying a self-reported questionnaire, containing questions with binary answers of the "yes" and "no” type. The questionnaire was organized in different sections that contained questions about socio demographic aspects, environmental, psychosocial, and behavioural factors, related to the prevention of mosquito-borne arboviruses. Each response was classified for one point if it was a preventive factor, or zero points otherwise. For example, each participant was asked whether they had covered water tanks at home. Having uncovered tanks is a risk factor because it favours the proliferation of mosquitoes; therefore, this response was classified as a risk, receiving zero points. The sum of points for each section defined a score, so that it was possible to evaluate a set of answers together. The more points, the more preventive behaviours people performed at the time the questionnaire was applied. The questionnaires were made available on the ZikaMob platform and answered by participants through their mobile devices. The validation of the questionnaire was described in the previous works [18, 19].

The *dependent variables* **(D)** correspond to the target behaviours of educational intervention, subdivided into two groups. To compose the **Target Behaviour Score (D),** participants were asked whether their water reservoirs, trash cans and capped drains were covered to avoid mosquitoes’ proliferation; whether their families survey reservoirs at least once a month, clean their water tanks, separate solid waste for recycling, inspect potted plants, close windows at dawn and dusk, and clean vacant lots. Each preventative behaviour performed received one point, so the score could vary from zero to eleven points. The **Breeding Identification and Elimination Score (DC),** ranging from zero to four points, was related to the behaviours reported by participants when identifying a mosquito breeding site. The participant had to know how to eliminate it; have learnt to throw water on sunny ground to eliminate the larvae; to wash containers; inspect other places in the residence for more breeding sites; to alert neighbours to the danger and to notify the Environmental Surveillance service about a prospective danger of infestation.

The *Independent variables* were grouped into subgroups. Sociodemographic variables consisted of gender, age, and role (student or teacher). Environmental and household risk were assessed through indicators such as: access to garbage collection services; running water and whether water was lacking two or more days a week; whether the household had a yard, plants, cistern, water tank or other water reservoirs that increase the risk of breeding; whether the residence was a single-storey house or building; and whether it was owned or rented. The higher the home or environmental risk in relation to having mosquito breeding sites, the higher the **Risk Score (R)** ranging from zero to 14 points.

**The Facilitator Behaviour Score (F)** refers to the fact that the participant already has some practices that may favour the accomplishment of the target behaviour, such as doing housework or taking care of potted plants or gardens. In order to compose the Perception of Prevalence Score **(P)**, ranging from 0 to 5 points, two levels of knowledge were assessed: 1) that of the participant (and, by extension, their family) about mosquito-borne arboviruses, and 2) their self-perception about diagnosing these diseases. Participants were asked whether they or their family members had had Zika, Dengue or Chikungunya; whether it is possible to get dengue more than once in their life; whether all mosquitoes transmit dengue fever and whether a dengue vaccine has already been developed.

Some constructs of behaviour change theories were also assessed by answering individual questions or adding points to the overall score. These are all grouped under the **T** score but can be broken down as follows: **The Self-Efficacy Score** assesses the self-efficacy and collective efficacy constructs that reveal whether a person believes they can perform the behaviour and change the behaviour of their family and neighbours. The constructs of the Health Belief Model (**Health Belief Score**) are concerned with the perception of the susceptibility, severity, barriers, and benefits associated with behaviour change. Each positive response meant having a certain belief or attitude that could favour preventative behaviour.

### Sample and Statistical Analysis

The study population was composed of all students and teachers of Campina Grande High Schools who agreed to participate in the project and signed the free and informed consent form. Out of a total of 3,681 students invited to participate voluntarily in this research, 883 (24%) students were included in this study because they answered at least one of the questionnaires used to assess attitudes, beliefs, and behaviours. Of this total, 227 participants answered the questionnaire before and after the intervention, and statistical tests were performed for paired samples to assess the change in behaviour of this group (Study A). An independent sample analysis was performed with a total of 626 participants, 364 of whom answered the questionnaire only before the intervention (Q1) and 262 who answered the questionnaire after the intervention (Q2) (Study B).

Descriptive statistics were used to describe the population profile and the frequency of each questionnaire response before and after the intervention. The normality test showed that the scores and age did not follow normal distribution, so only nonparametric tests were used for inferential analysis. Pearson's chi-square tests were used; Wilcoxon tests were also performed where paired samples were available, and Kruskal Wallis tests where there were independent samples (SIEGEL, 2006), both using the significance level of 5% (p-value <0.05). The reliability analysis of the instrument was performed using Cronbach's alpha, a test scorereliability coefficient for categorical variables. The analyses were performed with the aid of the R statistical software [21, 22].

In a second iteration of analysis, multivariate analysis techniques were used, adjusting the Principal Component Analysis (PCA), whose eigenvalues were greater than one (λ> 1), as suggested by Kaiser (1960), in order to identify a smaller number of variables: uncorrelated alternatives that somehow summarize the main information of the original variables. Subsequently, these main components were presented in Biplot graphs for individuals and variables with their respective confidence ellipses (with 95% reliability). Biplot is a method that represents two-dimensional multivariate data, where each observation is represented by the pair of scores of the first two main components, representing each group in their respective confidence ellipses. The PCA aimed to compare the patterns of these ellipses for the group of students and teachers, analysed before and after the intervention.

## Results

Of the 883 survey participants, 690 were students ranging in age from 14 to 41 years, with an average of 17.1 ± 2.5 years; and 193 were teachers from 22 to 64 years old, averaging 38.5 ± 9.2 years. In all, 510 (58%) participants were female: 393 students and 117 teachers (Table 1). Most participants (84%) owned a single-storey house with access to running water (97%), and only 13% reported lack of water for more than two days a week. In all, 34% reported using buckets to store water, 75% had water tanks and 21% had cisterns. Access to municipal waste collection services is virtually universal (96%). Of all households, 71% have a backyard where 51% of respondents grow plants or vegetable gardens. Around 55% of participants reported having abandoned houses or vacant lots nearby and 35% reported streams or sewers nearby their houses. On 64% of the land, there was trash that could serve as a mosquito breeding ground. In all, 37% of participants had unprotected roofs (i.e., no lining), potentially allowing mosquitoes to enter through the cracks in the tiles (Table 2).

**Table 1:** Descriptive analysis showing the profile of the population participating in the school-based intervention for arboviruses, performed with high school students from Campina Grande, Paraíba, Brazil

Participants		sex						age						
		Female		Male		Total		Average	SD	Min	Max	Median	P25	P75
		n	% valid	n	% valid	n	% valid							
Answered only Q1	Student	156	69,6%	94	67,1%	250	68,7%	16,6	1,3	14,0	22,0	17,0	16,0	17,0
	Teacher	68	30,4%	46	32,9%	114	31,3%	39,6	9,2	22,0	64,0	39,0	34,0	44,0
	Total	224	100,0%	140	100,0%	364	100,0%	23,8	11,9	14,0	64,0	17,0	16,0	33,0
Answered only Q2	Student	123	89,1%	114	91,9%	237	90,5%	18,0	3,8	14,0	41,0	17,0	16,0	18,0
	Teacher	15	10,9%	10	8,1%	25	9,5%	39,7	10,2	26,0	57,0	37,0	32,0	48,0
	Total	138	100,0%	124	100,0%	262	100,0%	20,0	8,0	14,0	57,0	17,0	16,0	19,0
Answered Q1 e Q2	Student	114	77,0%	89	81,7%	203	79,0%	16,5	1,2	14,0	21,0	16,0	16,0	17,0
	Teacher	34	23,0%	20	18,3%	54	21,0%	35,8	8,5	24,0	55,0	33,5	29,0	41,0
	Total	148	100,0%	109	100,0%	257	100,0%	20,6	8,8	14,0	55,0	17,0	16,0	18,0
**Student**		393	77,1%	297	79,6%	690	78,1%	17,1	2,5	14,0	41,0	17,0	16,0	18,0
**Teacher**		117	22,9%	76	20,4%	193	21,9%	38,5	9,2	22,0	64,0	37,5	32,0	44,0
Total		510	100,0%	373	100,0%	883	100,0%	21,7	10,1	14,0	64,0	17,0	16,0	20,0

*Abbreviations: Q1* Qquestionnaire before the intervention, *Q2* Questionnaire applied after the intervention, *n* number, *Valid%* Relative percentage of respondents, *SD* Standard deviation, *Min* Minimum value, *Max* Maximum value, *P25* 25th percentile or first quartile, *P75* 75th percentile or third quartile

**Table 2:** Results of school-based intervention for arboviruses, performed in the city of Campina Grande, Paraíba, Brazil. Frequency and percentage for each independent variable in the total population, and in the paired sample study (Study A)

Independent VARIABLES		Total Population *N*=883		Study A - Paired Analysis (*N*= 257)										
				Students (ST)					Teachers (TCH)					ST X TCH
				Pre		Post			Pre		Post			
		N	*%*	n	*%*	n	%	*p*	n	*%*	n	*%*	*p*	*p*
Gender	Fem	883	***58***	114	***44***	114	***44***	1	34	***13***	34	***13***	1	0.203
	Male		***42***	89	***35***	89	***35***		20	***8***	20	***8***		
R1 - Residence Type	House	848	***82***	179	***73***	179	***70***	0.23	37	***15***	37	***14***	0.519	<0.001*
	Apart.		***18***	16	***7***	24	***9***		12	***5***	16	***6***		
R2 – Homeowner	No	870	***16***	32	***13***	34	***13***	0.754	12	***5***	12	***5***	0.959	0.141
	Yes		***84***	171	***67***	167	***65***		41	***16***	42	***16***		
R3 - Piped water	No	880	***3***	3	***1***	2	***1***	0.653	1	***0***	0	***0***	0.32	0.799
	Yes		***97***	200	***78***	201	***79***		53	***21***	53	***21***		
R4 - Lack of water for more than two days a week	No	872	***13***	19	***7***	22	***9***	0.609	6	***2***	5	***2***	0.75	0.985
	Yes		***87***	184	***72***	180	***70***		48	***19***	49	***19***		
R5 - Water box	Yes	879	***75***	154	***60***	136	***54***	0.079	49	***19***	50	***20***	0.728	<0.001*
	No		***25***	49	***19***	64	***25***		5	***2***	4	***2***		
R6 – Tank	Yes	879	***21***	40	***16***	36	***14***	0.628	15	***6***	10	***4***	0.254	0.309
	No		***79***	163	***63***	166	***65***		39	***15***	44	***17***		
R7- Buckets or containers for storing water	No	877	***66***	166	***65***	156	***61***	0.221	20	***8***	31	***12***	0.042*	<0.001*
	Yes		***34***	37	***14***	47	***18***		33	***13***	23	***9***		
R8 - Access to garbage collection service	No	877	***4***	7	***3***	4	***2***	0.363	2	***1***	1	***0***	0.547	0.967
	Yes		***96***	195	***76***	197	***77***		51	***20***	53	***21***		
R9 - Household with yard	No	876	***71***	166	***66***	155	***61***	0.176	37	***15***	36	***14***	0.513	0.030*
	Yes		***29***	36	***14***	47	***18***		14	***6***	18	***7***		
R10 - Household with plants and vegetable garden	No	879	***49***	111	***43***	109	***43***	0.842	22	***9***	24	***9***	0.697	0.028*
	Yes		***51***	91	***36***	93	***36***		32	***13***	30	***12***		
R11 - Lined roof	No	867	***37***	106	***42***	86	***34***	**0.036**	13	***5***	13	***5***	0.912	<0.001*
	Yes		***63***	94	***37***	116	***45***		39	***15***	41	***16***		
R12 - Vacant land or abandoned houses near home	Yes	877	***55***	117	***46***	108	***42***	0.282	27	***11***	33	***13***	0.245	0.918
	No		***45***	82	***32***	94	***37***		27	***11***	21	***8***		
R13 - Garbage in the vacant lot	Yes	547	***64***	95	***63***	83	***54***	0.159	19	***13***	18	***12***	0.166	0.067
	No		***36***	29	***19***	38	***25***		8	***5***	16	***10***		
R14 - Streams and sewers near home	Yes	878	***35***	86	***34***	77	***30***	0.383	11	***4***	13	***5***	0.643	<0.001*
	No		***65***	116	***45***	124	***49***		43	***17***	41	***16***		
F1 - Helps with housework	No	877	***15***	39	***15***	40	***16***	0.9	4	***2***	1	***0***	0.169	<0.001*
	Yes		***85***	163	***64***	162	***63***		50	***20***	53	***21***		
F2 - Assists in the care of plants and gardens	No	663	***30***	69	***33***	64	***32***	0.842	4	***2***	2	***1***	0.434	<0.001*
	Yes		***70***	100	***48***	97	***49***		34	***16***	34	***17***		
F3 - Observed mosquitoes at home	No	879	***38***	65	***25***	89	***35***	0.14	15	***6***	25	***10***	0.46	0.865
	Yes		***62***	138	***54***	114	***44***		39	***15***	29	***11***		
F4 - It's important to do clean-up efforts	No	879	***2***	1	***0***	4	***2***	0.181	0	***0***	0	***0***	NC	0.248
	Yes		***98***	200	***79***	199	***77***		53	***21***	54	***21***		
P01 -Already had Zika, Dengue or Chikungunya	Yes	878	***36***	66	***26***	66	***26***	0.917	33	***13***	32	***12***	0.844	<0.001*
	Never		***64***	134	***53***	137	***53***		21	***8***	22	***9***		
P02 - Family members have had Zika, Dengue or Chikungunya	Yes	878	***46***	101	***40***	84	***33***	0.066	38	***15***	25	***10***	0.011*	0.022*
	No		***54***	99	***39***	119	***46***		16	***6***	29	***11***		
P03 - You get dengue more than once in your life	No	876	***11***	30	***12***	29	***11***	0.855	5	***2***	6	***2***	0.75	0.228
	Yes		***89***	170	***67***	173	***68***		49	***19***	48	***19***		
P04 - Every mosquito transmits dengue fever	Yes	879	***8***	8	***3***	10	***4***	0.653	2	***1***	2	***1***	1	0.729
	No		***92***	192	***76***	193	***75***		52	***20***	52	***20***		
P05- There is dengue vaccine	Yes	692	***39***	85	***46***	83	***38***	0.029*	6	***3***	4	***2***	0.459	<0.001*
	No		***61***	53	***29***	86	***40***		40	***22***	44	***20***		
T01- Arboviruses are severe and can lead to death	No	874	***3***	6	***2***	3	***1***	0.305	0	***0***	1	***0***	0.32	0.388
	Yes		***97***	194	***77***	199	***78***		53	***21***	53	***21***		
T02-Perceived risk of acquiring arboviruses	Low	866	***67***	148	***58***	145	***57***	0.617	29	***11***	34	***13***	0.329	0.003*
	High		***33***	52	***20***	57	***22***		25	***10***	20	***8***		
T03- Fear of acquiring arboviruses	No	879	***16***	41	***16***	35	***14***	0.403	2	***1***	3	***1***	0.647	<0.001*
	Yes		***84***	158	***62***	167	***65***		52	***21***	51	***20***		
T04 - Uses preventive measures	No	876	***7***	1	***0***	33	***13***	<0.001*	1	***0***	1	***0***	0.989	0.018*
	Yes		***93***	198	***79***	168	***66***		52	***21***	53	***21***		
T05 - Believes capable to change behaviours to reduce risk	No	878	***9***	18	***7***	26	***10***	0.214	1	***0***	2	***1***	0.558	0.009*
	Yes		***91***	182	***72***	176	***69***		53	***21***	52	***20***		
T06 - Believes family can change behaviours to reduce risk	No	876	***8***	18	***7***	20	***8***	0.758	3	***1***	1	***0***	0.308	0.052
	Yes		***92***	181	***72***	181	***71***		51	***20***	53	***21***		
T07 - Believe capable of convincing others to take preventive measures?	No	874	***16***	27	***11***	43	***17***	0.039*	3	***1***	7	***3***	0.184	0.037*
	Yes		***84***	172	***68***	158	***62***		51	***20***	47	***18***		
T08 - Believes family and neighbours can change lifestyle habits	No	879	***12***	17	***7***	33	***13***	0.019*	2	***1***	7	***3***	0.082	0.236
	Yes		***88***	182	***72***	170	***66***		52	***21***	47	***18***		

Pearson's chi-square test was performed comparing students (ST) and teachers (TCH), independently, before and after the intervention. The analysis was also made by comparing the frequencies in the group of students and teachers (ST x TCH)

*Abbreviations: N* Absolute population number, *n* Number in the sample, % Percentage of valid responses excluding missing data

The frequencies and results of Pearson's chi-square test on household and environmental risk variables for Study A (paired sample) were shown in Table 2, comparatively before and after intervention, respectively. No significant differences were found in the paired sample, indicating excellent reliability because the same participants answered identical questions before and after the intervention (Table 2). The Cronbach's alpha result for all categorical variables was 0.966, indicating excellent internal validity of the questionnaire used.

Regarding the comparison between students and teachers, a quite different pattern was found between the two groups in seven of the 14 variables. Teachers have greater financial stability and live more commonly in a well-built building and therefore have a lower risk of mosquito breeding in their homes (Table 2). In the study B (unpaired sample), more male students living in well-built buildings responded to Q2, giving rise to different risk conditions such as access to or lack of water (Supplementary Table 1).

In all, 85% of respondents reported helping with household chores; 75% assisting in the care of plants and gardens; 62% have observed mosquitoes in their homes, and 98% believe that it is important to make efforts to clean up waste land and homes to reduce the prevalence of mosquito-borne arboviruses (Table 2). Teachers do more housework and care more for plants and gardens than students do (Table 2). Results for the unpaired sample show differences in all facilitating behaviours in the student group, although the two data collection points may have different results because more men responded to Q2 (Supplementary Table 1) than had to Q1.

Of all participants, 36% said they had had dengue, zika or chikungunya. In both samples, there was a significant reduction in the self-reported prevalence of these diseases for family members after the intervention (Table 2). In all, 11% of participants mistakenly believe that dengue infection can occur only once in a lifetime and 8% stated that there is a vaccine available for this disease. Almost all participants (97%) recognized that dengue can lead to death and 84% were afraid of acquiring one of these diseases; however most (67%) believed that their risk of infection is low because they adopt preventive measures (97%) (Table 2).

In the paired sample, there was no significant difference in these perceptions before and after the intervention, except for preventive measures. One noteworthy feature was that teachers showed greater fear of acquiring these diseases (*p*<0.001), with significantly different perceptions of susceptibility and severity than students (*p*=0.003) (Table 2). In the unpaired sample, there were clear differences in students' knowledge with the intervention (i.e., there was a bigger pre-post difference to that shown by teachers) and there were varying degrees of difference in relation to all constructs when comparing teachers and students, reproducing the findings of the paired sample (Supplementary Table 1).

Regarding self-efficacy, over 90% of participants believed they were able to change their behaviour, and 84% felt able to change the behaviours of their families, friends, and neighbours (Table 2). Teachers believed more than students in their ability to convince others to change their behaviour (*p*=0.037). In the paired sample, contrastingly, after the intervention, participants reported a lower conviction in their ability to change (*p*=0.039), although they came to believe more that they could alter the practices of relatives and neighbours (*p*=0.019) (Table 2). In the unpaired sample, there was a significant difference between students and teachers for almost all responses except the belief in changing neighbours and family members (Supplementary Table 1).

### Behaviour change

Table 3 shows the frequencies of target behaviours and chi-square test results comparing groups of students and teachers, before and after the intervention. In Study A, a significant difference was found in relation to seven of the 16 behaviours that were targeted by the intervention: about ten per cent more participants began household surveys, donating recyclables to pickers, screening windows and drains, and closing doors and windows at dawn and dusk. When identifying a breeding site, after the intervention, participants pointed to the need to survey other places in the residence and alert the Environmental Surveillance service (Table 3). When compared to teachers, students began to separate more solid waste for recycling (*p*=0.002), with a 7% increase in the performance of this action after the intervention (Table 3).

**Table 3:** Results of school-based intervention for arboviruses, performed in the city of Campina Grande, Paraíba, Brazil. Frequency and percentage for each dependent variable in the total population, and in the paired sample study (Study A)

Dependent Variables		Total Population		Study A - Paired Analysis (N=257)										
				Students (ST)					Teachers (TCH)					ST X TCH
		*N*=883		Pre		Post		*p*	Pre		Post		*p*	*p*
		N	*%*	n	*%*	n	*%*		n	*%*	n	*%*		
D1 - Water reservoirs stay open	Yes	651	***10***	19	***10***	13	***7***	0.313	1	***1***	1	***1***	0.761	0.121
	No		***90***	149	***78***	149	***76***		34	***17***	22	***12***		
D2 - Family survey reservoirs at least once a month	No	784	***23***	47	***19***	30	***14***	0.124	4	***2***	14	***6***	0.026*	0.922
	Yes		***77***	149	***61***	142	***67***		36	***17***	34	***14***		
D3 - Family cleans water tanks	No	753	***14***	29	***12***	26	***12***	0.865	3	***1***	12	***5***	0.051	0.729
	Yes		***86***	156	***66***	147	***70***		35	***17***	39	***17***		
D4 -Open trash cans	Yes	878	***20***	43	***17***	48	***19***	0.552	7	***3***	15	***6***	0.062	0.681
	No		***80***	160	***62***	155	***61***		46	***18***	39	***15***		
D5 -Family separates solid waste for recycling	No	877	***56***	146	***57***	128	***50***	0.075	27	***11***	29	***11***	0.70	0.002*
	Yes		***44***	56	***22***	72	***28***		27	***11***	25	***10***		
D6 - Family donates recyclables to waste pickers	No	876	***49***	119	***46***	102	***40***	0.080	22	***9***	30	***12***	0.123	0.235
	Yes		***51***	83	***32***	101	***39***		32	***12***	24	***9***		
D7-Family inspects potted plants	No	637	***21***	37	***19***	34	***18***	0.846	2	***1***	1	***1***	0.555	0.001*
	Yes		***79***	126	***64***	122	***65***		31	***16***	32	***16***		
D8-Windows with screens	No	877	***74***	174	***68***	147	***58***	0.001*	41	***16***	46	***18***	0.224	0.835
	Yes		***26***	28	***11***	54	***21***		13	***5***	8	***3***		
D9- Family usually close windows at dawn and dusk	No	880	***24***	54	***21***	30	***12***	0.004*	11	***4***	20	***8***	0.056	0.078
	Yes		***76***	149	***58***	172	***67***		43	***17***	34	***13***		
D10-Capped Drains	No	879	***20***	48	***19***	31	***12***	0.035*	8	***3***	14	***5***	0.152	0.841
	Yes		***80***	155	***60***	171	***67***		46	***18***	40	***16***		
D11- Family usually cleans vacant lots	No	618	***66***	98	***59***	102	***51***	0.171	32	***16***	22	***13***	0.939	0.938
	Yes		***34***	35	***21***	52	***26***		14	***7***	10	***6***		
DC0- Breeding site - have found mosquito larvae in your home	Yes	880	***47***	109	***43***	102	***40***	0.171	21	***8***	24	***9***	0.939	0.048*
	No		***53***	93	***36***	99	***39***		33	***13***	30	***12***		
DC1- When finding the breeding site- made or would dispose of water on land and in sunny location	No	866	***19***	37	***15***	37	***14***	0.518	9	***4***	10	***4***	0.558	0.885
	Yes		***81***	164	***65***	165	***64***		45	***18***	43	***17***		
DC2- When finding the breeding site - did or would sanitize with bleach and bushing	No	867	***6***	14	***5***	6	***2***	0.981	3	***1***	6	***2***	766	0.169
	Yes		***94***	187	***73***	197	***77***		50	***20***	48	***19***		
DC3- When finding the breeding site - did or would do inspection of the house	No	876	***4***	3	***1***	13	***5***	0.063	1	***0***	0	***0***	0.310	0.117
	Yes		***96***	197	***78***	190	***74***		53	***21***	54	***21***		
DC4- Finding the breeding site - warned or would warn neighbors	No	869	***4***	6	***2***	7	***3***	0.012*	1	***0***	0	***0***	0.315	0.190
	Yes		***96***	193	***76***	194	***76***		53	***21***	54	***21***		
DC5- Upon finding the breeding site, notified or would notify the Environmental Surveillance service	No	870	***46***	108	***43***	87	***34***	0.792	13	***5***	21	***8***	0.315	0.001*
	Yes		***54***	91	***36***	114	***45***		41	***16***	33	***13***		

Pearson's chi-square test was performed comparing students (ST) and teachers (TCH), independently, before and after the intervention. The analysis was also made by comparing the frequencies in the group of students and teachers (ST x TCH)

*Abbreviations: N* Absolute population number, *n* Number in the sample, % Percentage of valid responses excluding missing data

In study B (unpaired sample), significant differences were observed in relation to 10 of the 16 target arboviral prevention behaviours. Fewer water reservoirs were opened, and families began to survey more often. Households both separated more recyclables and donated them to waste pickers after the intervention. One of the most significant changes concerns window screening. In the student group, positive responses rose from 9% before missions to 49% after the intervention (Supplementary Table 2).

### Comparison of scores

Table 4 shows the results of the Wilcoxon test for the paired sample, comparing the median values for the scores before and after the intervention. We found that there was a significant difference in almost all scores, except in the measures of changes in the perceptions of susceptibility, perceived risk, and severity of arboviruses (*p*=0.125). This means that the intervention promoted changes in perceptions, attitudes, and behaviours except those associated with the Health Belief Model.

**Table 4:** Descriptive and inferential analysis of the scores for a paired and unpaired sample of the school-based intervention for arboviruses, performed in the city of Campina Grande, Paraíba, Brazil

Studies		Study A - Scores - Paired Analysis (*N*=257)											Study B - Scores - Unpaired Analysis (*N*=626)											
Phase		Pre Intervention					Post Intervention					Wilcoxon Test	Pre Intervention					Post Intervention					Kruskal-Wallis Test	
		Min	Max	MD	Quartiles		Min	Max	MD	Quartiles		PRE X POST	Min	Max	MD	Quartiles		Min	Max	MD	Quartiles		ST X TCH	PRE X POST
Scores					1^st^	3^rd^				1^st^	3^rd^	*p*-value				1^st^	3^rd^				1^st^	3^rd^	(*p*-value)	(*p*-value)
**Risk score**	ST	3	12	7	6	8	3	14	7	6	9	0.001*	3	12	7	6	8	3	14	7	6	9	0.007*	0.163
	TCH	6	12	8	7	9	5	12	8	7	10		6	12	8	7	9	5	12	8	7	10		
	TOT	**3**	**12**	**7**	**6**	**8**	**3**	**14**	**8**	**6**	**9**		**3**	**12**	**7**	**6**	**8**	**3**	**14**	**8**	**6**	**9**		
**Target behavior score**	ST	1	11	6	5	8	1	11	7	5	8	<0.001*	1	11	6	5	8	1	11	7	5	8	<0.001*	<0.001*
	TCH	0	10	6	4	8	1	10	6,5	5	8		0	10	6	4	8	1	10	6,5	5	8		
	TOT	**0**	**11**	**6**	**5**	**8**	**1**	**11**	**7**	**5**	**8**		**0**	**11**	**6**	**5**	**8**	**1**	**11**	**7**	**5**	**8**		
**Breeding Identification And Elimination Score**	ST	0	6	5	4	5	1	6	5	4	5	0.021*	0	6	5	4	5	1	6	5	4	5	**0.037**	0.033*
	TCH	2	6	5	4	6	3	6	5	5	6		2	6	5	4	6	3	6	5	5	6		
	TOT	**0**	**6**	**5**	**4**	**5**	**1**	**6**	**5**	**4**	**6**		**0**	**6**	**5**	**4**	**5**	**1**	**6**	**5**	**4**	**6**		
**Perception Of Prevalence Score**	ST	0	5	3	3	4	0	5	4	3	4	<0.001*	0	5	3	3	4	0	5	4	3	4	0.211	<0.001*
	TCH	1	5	3	3	4	1	5	4	3	5		1	5	3	3	4	1	5	4	3	5		
	TOT	**0**	**5**	**3**	**3**	**4**	**0**	**5**	**4**	**3**	**4**		**0**	**5**	**3**	**3**	**4**	**0**	**5**	**4**	**3**	**4**		
**Facilitator Behavior Score**	ST	1	4	3	2	4	1	4	3	2	4	0.023*	1	4	3	2	4	1	4	3	2	4	0.196	0.639
	TCH	1	4	3	3	4	1	4	3	3	4		1	4	3	3	4	1	4	3	3	4		
	TOT	**1**	**4**	**3**	**3**	**4**	**1**	**4**	**3**	**2**	**4**		**1**	**4**	**3**	**3**	**4**	**1**	**4**	**3**	**2**	**4**		
**Health Belief Score**	ST	0	4	3	3	3	1	4	3	2	3	0.125	0	4	3	3	3	1	4	3	2	3	<0.001*	0.494
	TCH	2	4	3	3	4	1	4	3	3	4		2	4	3	3	4	1	4	3	3	4		
	TOT	**0**	**4**	**3**	**3**	**4**	**1**	**4**	**3**	**3**	**3**		**0**	**4**	**3**	**3**	**4**	**1**	**4**	**3**	**3**	**3**		
**Self-efficiency score**	ST	0	4	4	3	4	0	4	4	3	4	0.017*	0	4	4	3	4	0	4	4	3	4	**0.001**	0.683
	TCH	1	4	4	4	4	1	4	4	4	4		1	4	4	4	4	1	4	4	4	4		
	TOT	**0**	**4**	**4**	**4**	**4**	**0**	**4**	**4**	**3**	**4**		**0**	**4**	**4**	**4**	**4**	**0**	**4**	**4**	**3**	**4**		

*Abbreviations: MD* Median, *Min* Minimum value, *Max* Maximum value, *ST* Student, *TCH* Teachers, *Tot* Total

In the unpaired sample, for which the Kruskal Wallis test was used, a significant difference was observed in the environmental and home risk scores between students and teachers (*p*=0.007) (Table 4). A significant difference was also observed between teachers and students in relation to prevention behaviour target scores (*p*<0.001) and breeding stock elimination (*p*=0.037), and for knowledge and perceptions regarding the prevalence and manifestation of arboviruses (*p*<0.001).

Teachers and students also showed significant differences regarding preventative behaviours, perceptions of risk and susceptibility and self-efficacy. In addition, teachers were more afraid of acquiring arboviruses (*p*<0.001) and less confident of being able to convince others to take preventive measures (*p*=0.001) (Table 4).

### Multivariate analysis

**Fig. 1:**
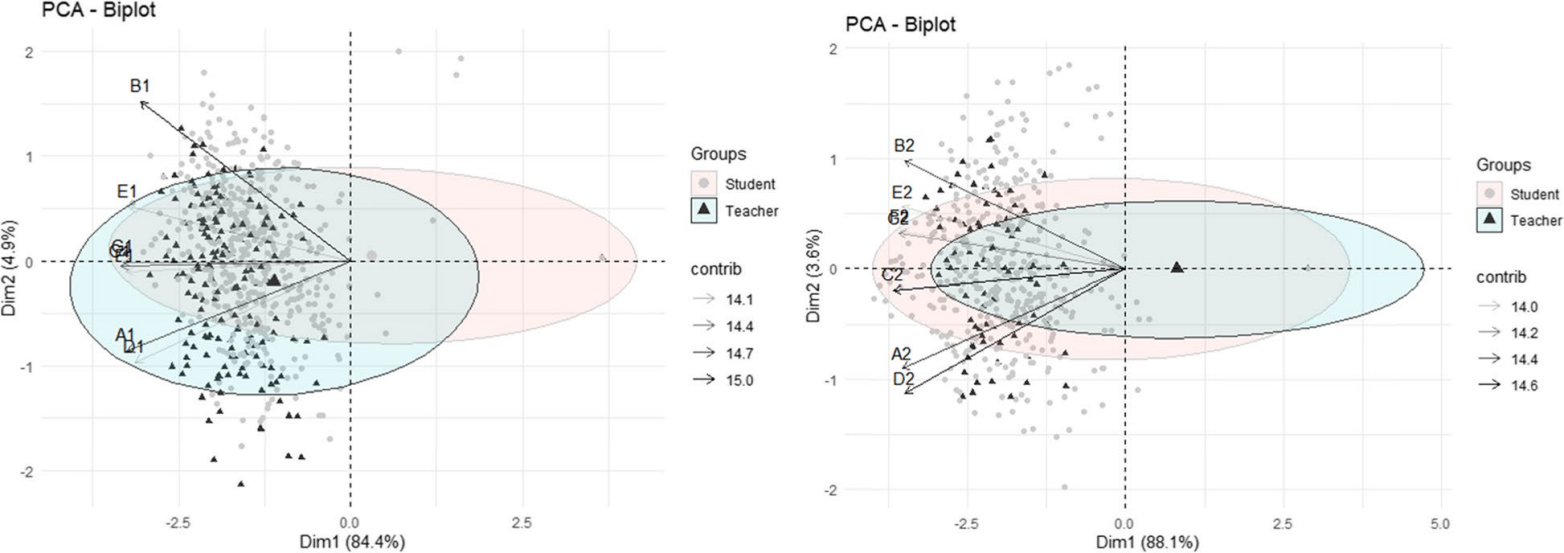
Multivariate analysis showing Biplot plots for individuals and groups (High School Students and High School Teachers) with their respective confidence ellipses (with 95% reliability). Variables with number 1 refer to the pre-intervention data analysis and with number 2 refer to the post-intervention for arboviruses prevention. Abbreviations: A – Risk Score; B – Target Behaviour Score; C – Breeding Identification and Elimination Score; D – Perception of Prevalence Score; E – Facilitator Behaviour Score; F – Health Belief Score and G – Self-efficacy Score

Figure 1 represents the Biplot graph resulting from the Principal Component Analysis (PCA) showing the patterns for the group of students and teachers, analysed before and after the intervention. Both groups showed similar patterns regarding prevention of arboviruses. Following the intervention, it was found that students had appropriated the requisite knowledge, perceptions, and behaviours in such a way as to have greater overlap with the group of teachers.

## Discussion

This study showed that educational interventions using a virtual platform can promote behavioural change regarding arbovirus prevention, corroborating the findings of preliminary studies conducted with smaller samples [18, 19]. The use of the ZikaMob platform adapted for mobile devices facilitated effective teaching about arboviruses and their prevention, better organization against their spread, and greater performance of synchronous preventive actions involving students from different schools and neighbourhoods of the second most populous city of Paraíba, in northeastern Brazil. This is an innovative strategy that has the potential to be replicated in any location that has an internet network and can involve an unlimited number of participants as long as they have a mobile device. This study corroborates findings from the literature that describe interventions based on behaviour change theories [23] and use of digital technologies [24].

In Brazil, dengue prevention guidelines and policies have set social mobilization goals and directed calls for action at schools; however, they have not defined strategies for carrying them out. In this paper, for the first time in Brazil, we described the development of a platform that allows the inclusion of all students and teachers in a city in order to perform synchronized prevention actions which are disseminated through social networks, increasing the number of people affected by the actions. A systematic literature review has shown that complex population-oriented interventions are more effective in reducing vector mosquitoes than specific actions [25]. This study has also shown that multifaceted interventions involving the community and professionals from the fields of health, education, and the wider social infrastructure, including the mass media, are more likely to be effective for vector control.

Arbovirus vector mosquitoes proliferate during the rainy season [26, 27]; for this reason, preventive actions must be planned and implemented at certain times of the year in order to maximize their effects. Using the strategy described in this paper may facilitate the synchronization of these actions and allow for the establishment of a shared calendar between schools and health services, enhancing results and reducing costs related to human and material resources. The results of this work showed a clear change in population behaviour with the use of school interventions. Therefore, the revision of public policies related to vector control is recommended, in order to incorporate the model and strategies described herein.

In this project, the researchers publicized the project in all high schools in the city of Campina Grande; however, engagement was dependent on the individual and voluntary decision of teachers and school principals. To reward the teachers’ efforts and motivate them to participate in the preventive actions, an 80-hour continuing education course certificate was given to the participants that could be used as part of salary bonus requirements. Of the 490 teachers who registered, only 193 performed the proposed activities on the virtual platform, but there is a clear correlation: in schools where teachers engaged, more students also participated in the actions.

The mobilization and engagement of teachers, students, and the community in interventions to prevent arboviruses needs to be understood as a collective project and as public policy, with technical guidelines, timing and integrated management. Teacher dropout occurred because prevention actions were not part of the school's calendar of activities and were not considered a priority by the managers. These preventive actions were not foreseen in the action plans of schools or health services.

One of the barriers to the integration of preventive actions with arboviruses in Brazil is the lack of agreement between federative entities. Surveillance and environmental education services are the responsibility of municipalities, and the management of high schools is the responsibility of the state. Due to political differences, it is often difficult to develop joint action between municipal and state managers. In addition, as has occurred in other Latin American countries, there is a growth in urban violence that has hampered entomological surveillance actions [28]. In Campina Grande, health workers reported that they were unable to enter up to 70% of households for inspection because residents were working or did not allow them to enter for fear of being robbed [11]. The authors contend that, in urban regions, the traditional model of home surveillance performed by health workers in the context of urban violence should be investigated further and include an assessment of cost-effectiveness [11].

There is evidence in the literature of randomized trials showing the effectiveness of using window screens with or without insecticides [29], mosquito nets or curtains [30] to reduce the prevalence of dengue. In Brazil, the guidelines for vector control did not prioritize the use of screens on windows and doors, either with or without insecticides, as a mechanical barrier method to avoid direct contact with mosquitoes. Instead, vector control actions have been based on the use of larvicides and insecticides [31, 32], which has led to an increase in resistant mosquito populations [33]. In this paper, we found that the population was unaware of these preventive measures because they are not publicized in traditional government campaigns; and that there was a significant difference in the use of window screens due to the intervention.

Our outcomes showed that intervention improved recycling. The separation of solid waste with a donation to waste pickers is one of the behaviours that contribute to reducing social inequalities and improving the environment and the health of populations [34]. Plastics dispensed in inappropriate places such as gardens and open land serve as breeding grounds for mosquitoes [34]. In Brazil, the garbage is placed in plastic bags at the gates of households without separation of recyclables, as most cities do not offer selective collection service. Waste pickers, usually illiterate people in socially vulnerable situations, open their bags on the streets and take advantage of recyclables, often leaving the garbage scattered. When the population separates the recyclables, this increases the income of the pickers. In this paper, a strategy to articulate the actions of entomological control to those of recycling was evidenced. By sorting and donating recyclables to waste pickers, people see a reward or benefit in changing their behaviours. To date, this was the first intervention study to establish the link between home inspection and recycling actions.

Most of the participants in this study reported fear of acquiring arboviruses, but the perceived risk is low, corroborating findings from another study in French Guiana [4, 35]. Students showed lower risk perception than their teachers or health workers [19]. Younger individuals tend to have lower risk perception and lower concern about acquiring arboviruses [4, 35, 36]; as do women of reproductive age [37]. Risk is higher among pregnant women than non-pregnant women [38, 39] and there is also a higher rate of disease acquisition in this group [40]. Living in an area with a higher prevalence of the disease does not change the perception of risk and susceptibility [35, 40].

In Pakistan, logistic regression analysis showed that perceived risk and self-efficacy are predictors of dengue prevention practices [40]. Regarding self-efficacy, we found that the participants in this study believe they can change their behaviours, and those of their family members, colleagues and neighbours. With the intervention, participants began to reflect more on their ability to convince others to take collective preventive actions and the use of social networks to accomplish this.

Most studies on arboviruses in the literature describe perceptions and knowledge about arboviruses prevention [41, 42], with few reports of school-based interventions [43–45]. Most of these studies, however, are not randomized case-control trials based on behaviour change theories that can offer evidence of intervention impact and effectiveness [39]. In Puerto Rico, for example, an intervention by the Department of Health in partnership with the Centre for Diseases Control and Prevention (CDC) from the United States was performed, which showed a significant impact on knowledge and preventive behaviour for dengue, and reduced rates of mosquito infestation [39, 46].

The limitations of this study concern firstly the fact that the questionnaire is self-reported and secondly the design of the study itself. However, the paired analysis showed that our data were reliable. To verify the impact of interventions, the ideal design would be a case-control study; however, in this work, teachers and students receive a reward (prize) for participating in the research; therefore, there was no way to create a “control group”. In addition, there is the possibility of using the *Aedes aegypti* Rapid Infestation Index (LIRAa) [20] that measures the level of vector mosquito infestation in urban strata (territorial units with 10,000 inhabitants) to assess the impact of educational intervention. These measures are performed by health workers three times a year and could be used to assess the impact of educational activities. However, to carry out this type of study it would be necessary for all high school students from Campina Grande to participate in the intervention, georeferencing these students and reducing the size of the LIRAa territorial unit and / or the joint definition of research involving the services of Environmental Surveillance and the Secretariat of Education [20]. Moreover, health workers should be integrated into educational activities. During the intervention research, their activities were mostly paralysed due to a strike.

## Conclusion

The findings of this work show that school-based interventions can promote change in attitudes and behaviours in the population, which could lead to a reduction in infestation and a lower risk of illness and death from arbovirus.

## Supplementary Information

**Additional file 1.**

**Additional file 2.**

## Data Availability

The datasets used and/or analysed during the current study are available from the corresponding author on reasonable request.
