# Supplementary material for: Prevention and control of mosquito-borne arboviral diseases: lessons learned from a school-based intervention in Brazil (Zikamob)
Source: BMC Public Health. 2022 Feb 8;22:255. doi: 10.1186/s12889-022-12554-w (PMC8822808; doi:10.1186/s12889-022-12554-w)
Supplement: Supplementary file 1 — Additional file 1. [file 12889_2022_12554_MOESM1_ESM.docx]

Supplementary Table 1 - Results of school-based intervention for arboviruses, performed in the city of Campina Grande, Paraíba, Brazil. Frequency and percentage for each independent variable in unpaired sample (Study B). Pearson's chi-square test was performed comparing students (ST) and teachers (TCH), independently, before and after the intervention. The analysis was also made by comparing the frequencies in the group of students and teachers (ST x TCH). Abbreviations: N - absolute population number; n - number in the sample; % - percentage of valid responses excluding missing data.

| **INDEPENDENT VARIABLES** | | **STUDY B - UNPAIRED ANALYSIS (N= 626)** | | | | | | | | | | |
| --- | --- | --- | --- | --- | --- | --- | --- | --- | --- | --- | --- | --- |
|  |  | **Students (ST)** | | | | | **Teachers (TCH)** | | | | | **ST X TCH** |
|  |  | **Pre** | | **Post** | |  | **Pre** | | **Post** | |  |  |
|  |  | **n** | ***%*** | **N** | ***%*** | **p** | **n** | ***%*** | **n** | ***%*** | **p** | **p** |
| Gender | Fem | 156 | ***43*** | 123 | ***47*** | **0.019*** | 68 | ***19*** | 15 | ***6*** | 0.974 | 0.61 |
|  | Male | 94 | ***26*** | 114 | ***44*** |  | 46 | ***13*** | 10 | ***4*** |  |  |
| R1 - Residence Type | House | 225 | ***65*** | 167 | ***68*** | **<0.001*** | 71 | ***21*** | 18 | ***7*** | 0.717 | **<0.001*** |
|  | Apart. | 17 | ***5*** | 54 | ***22*** |  | 33 | ***10*** | 7 | ***3*** |  |  |
| R2 – Homeowner | No | 30 | ***8*** | 40 | ***16*** | 0.08 | 19 | ***5*** | 3 | ***1*** | 0.563 | 0.744 |
|  | Yes | 220 | ***60*** | 186 | ***74*** |  | 95 | ***26*** | 22 | ***9*** |  |  |
| R3 - Piped water | No | 7 | ***2*** | 14 | ***5*** | 0.09 | 1 | ***0*** | 0 | ***0*** | 0.645 | **0.043*** |
|  | Yes | 243 | ***67*** | 222 | ***85*** |  | 113 | ***31*** | 24 | ***9*** |  |  |
| R4 - Lack of water for more than two days a week | No | 29 | ***8*** | 49 | ***19*** | **0.007*** | 8 | ***2*** | 3 | ***1*** | 0.39 | **0.018*** |
|  | Yes | 217 | ***61*** | 186 | ***72*** |  | 103 | ***29*** | 21 | ***8*** |  |  |
| R5 - Water box | Yes | 177 | ***49*** | 170 | ***65*** | 0.764 | 98 | ***27*** | 24 | ***9*** | 0.165 | **<0.001*** |
|  | No | 73 | ***20*** | 66 | ***25*** |  | 16 | ***4*** | 1 | ***0*** |  |  |
| R6 – Tank | Yes | 53 | ***15*** | 58 | ***22*** | 0.362 | 21 | ***6*** | 7 | ***3*** | 0.234 | 0.518 |
|  | No | 197 | ***54*** | 177 | ***68*** |  | 93 | ***26*** | 17 | ***7*** |  |  |
| R7- Buckets or containers for storing water | No | 184 | ***51*** | 144 | ***56*** | **0.004*** | 50 | ***14*** | 16 | ***6*** | 0.74 | **<0.001*** |
|  | Yes | 65 | ***18*** | 89 | ***34*** |  | 63 | ***17*** | 9 | ***3*** |  |  |
| R8 - Access to garbage collection service | No | 16 | ***4*** | 13 | ***5*** | 0.679 | 2 | ***1*** | 1 | ***0*** | 0.461 | 0.073 |
|  | Yes | 233 | ***64*** | 222 | ***86*** |  | 112 | ***31*** | 23 | ***9*** |  |  |
| R9 - Household with yard | No | 199 | ***55*** | 150 | ***58*** | **<0.001*** | 74 | ***20*** | 12 | ***5*** | 0.171 | **0.022** |
|  | Yes | 48 | ***13*** | 85 | ***33*** |  | 40 | ***11*** | 12 | ***5*** |  |  |
| R10 - Household with plants and vegetable garden | No | 136 | ***37*** | 102 | ***39*** | **0.011*** | 52 | ***14*** | 11 | ***4*** | 0.846 | 0.537 |
|  | Yes | 113 | ***31*** | 135 | ***52*** |  | 62 | ***17*** | 12 | ***5*** |  |  |
| R11 - Lined roof | No | 122 | ***34*** | 72 | ***29*** | **<0.001*** | 21 | ***6*** | 6 | ***2*** | 0.537 | **<0.001*** |
|  | Yes | 126 | ***35*** | 153 | ***61*** |  | 92 | ***25*** | 19 | ***8*** |  |  |
| R12 - Vacant land or abandoned houses near home | Yes | 137 | ***38*** | 103 | ***40*** | 0.14 | 85 | ***23*** | 18 | ***7*** | 0.791 | **<0.001*** |
|  | No | 111 | ***31*** | 131 | ***51*** |  | 29 | ***8*** | 7 | ***3*** |  |  |
| R13 - Garbage in the vacant lot | Yes | 100 | ***44*** | 90 | ***55*** | **0.090*** | 51 | ***22*** | 10 | ***6*** | 0.701 | 0.671 |
|  | No | 44 | ***19*** | 60 | ***36*** |  | 32 | ***14*** | 5 | ***3*** |  |  |
| R14 - Streams and sewers near home | Yes | 81 | ***22*** | 86 | ***33*** | 0.347 | 41 | ***11*** | 8 | ***3*** | 0.707 | 0.87 |
|  | No | 168 | ***46*** | 149 | ***57*** |  | 73 | ***20*** | 17 | ***7*** |  |  |
| F1 - Helps with housework | No | 51 | ***14*** | 28 | ***11*** | **0.011*** | 9 | ***2*** | 3 | ***1*** | 0.467 | **0.025*** |
|  | Yes | 198 | ***55*** | 206 | ***80*** |  | 105 | ***29*** | 21 | ***8*** |  |  |
| F2 - Assists in the care of plants and gardens | No | 82 | ***31*** | 36 | ***18*** | **<0.001*** | 12 | ***5*** | 2 | ***1*** | 0.665 | **0.005*** |
|  | Yes | 113 | ***42*** | 148 | ***74*** |  | 59 | ***22*** | 14 | ***7*** |  |  |
| F3 - Observed mosquitoes at home | No | 80 | ***22*** | 99 | ***38*** | **0.030*** | 31 | ***9*** | 14 | ***5*** | **0.006*** | 0.382 |
|  | Yes | 168 | ***47*** | 138 | ***53*** |  | 81 | ***23*** | 11 | ***4*** |  |  |
| F4 - It's important to do clean-up efforts | No | 3 | ***1*** | 12 | ***5*** | **0.013*** | 0 | ***0*** | 1 | ***0*** | **0.033*** | 0.12 |
|  | Yes | 246 | ***68*** | 223 | ***86*** |  | 113 | ***31*** | 24 | ***9*** |  |  |
| P01 -Already had Zika, Dengue or Chikungunya | Yes | 89 | ***25*** | 61 | ***23*** | **0.020*** | 54 | ***15*** | 16 | ***6*** | 0.153 | **<0.001*** |
|  | Never | 160 | ***44*** | 174 | ***67*** |  | 58 | ***16*** | 9 | ***3*** |  |  |
| P02 - Family members have had Zika, Dengue or Chikungunya | Yes | 127 | ***35*** | 90 | ***34*** | **0.004*** | 60 | ***17*** | 16 | ***6*** | 0.343 | **0.028** |
|  | No | 121 | ***34*** | 146 | ***56*** |  | 52 | ***14*** | 9 | ***3*** |  |  |
| P03 - You get dengue more than once in your life | No | 33 | ***9*** | 26 | ***10*** | 0.452 | 3 | ***1*** | 2 | ***1*** | 0.2 | **0.04** |
|  | Yes | 215 | ***60*** | 209 | ***80*** |  | 109 | ***30*** | 23 | ***9*** |  |  |
| P04 - Every mosquito transmits dengue fever | Yes | 18 | ***5*** | 35 | ***13*** | **0.007*** | 5 | ***1*** | 2 | ***1*** | 0.454 | **0.037** |
|  | No | 230 | ***64*** | 200 | ***77*** |  | 109 | ***30*** | 23 | ***9*** |  |  |
| P05- There is dengue vaccine | Yes | 111 | ***41*** | 59 | ***29*** | **<0.001*** | 12 | ***4*** | 1 | ***0*** | 0.266 | **<0.001*** |
|  | No | 65 | ***24*** | 125 | ***61*** |  | 81 | ***30*** | 21 | ***10*** |  |  |
| T01- Arboviruses are severe and can lead to death | No | 12 | ***3*** | 9 | ***3*** | 0.594 | 0 | ***0*** | 0 | ***0*** | NC | **0.012*** |
|  | Yes | 235 | ***65*** | 224 | ***87*** |  | 113 | ***31*** | 25 | ***10*** |  |  |
| T02-Perceived risk of acquiring arboviruses | Low | 185 | ***51*** | 137 | ***55*** | **0.003*** | 61 | ***17*** | 15 | ***6*** | 0.421 | **0.004*** |
|  | High | 64 | ***18*** | 86 | ***35*** |  | 53 | ***15*** | 9 | ***4*** |  |  |
| T03- Fear of acquiring arboviruses | No | 49 | ***14*** | 42 | ***16*** | 0.566 | 7 | ***2*** | 3 | ***1*** | 0.311 | **0.001*** |
|  | Yes | 199 | ***55*** | 195 | ***74*** |  | 106 | ***29*** | 22 | ***8*** |  |  |
| T04 - Uses preventive measures | No | 2 | ***1*** | 25 | ***10*** | **<0.001*** | 0 | ***0*** | 1 | ***0*** | **0.033*** | **0.015*** |
|  | Yes | 246 | ***68*** | 210 | ***81*** |  | 113 | ***31*** | 24 | ***9*** |  |  |
| T05 - Believes capable to change behaviours to reduce risk | No | 25 | ***7*** | 18 | ***7*** | 0.35 | 3 | ***1*** | 1 | ***0*** | 0.711 | **0.018*** |
|  | Yes | 223 | ***62*** | 217 | ***83*** |  | 111 | ***31*** | 24 | ***9*** |  |  |
| T06 - Believes family can change behaviours to reduce risk | No | 28 | ***8*** | 18 | ***7*** | 0.179 | 2 | ***1*** | 1 | ***0*** | 0.484 | **0.004*** |
|  | Yes | 220 | ***61*** | 216 | ***83*** |  | 112 | ***31*** | 24 | ***9*** |  |  |
| T07 - Believe capable of convincing others to take preventive measures? | No | 47 | ***13*** | 37 | ***14*** | 0.342 | 4 | ***1*** | 6 | ***2*** | **<0.001*** | **0.004*** |
|  | Yes | 200 | ***56*** | 198 | ***76*** |  | 108 | ***30*** | 19 | ***7*** |  |  |
| T08 - Believes family and neighbours can change lifestyle habits | No | 25 | ***7*** | 28 | ***11*** | 0.53 | 10 | ***3*** | 1 | ***0*** | 0.418 | 0.31 |
|  | Yes | 223 | ***62*** | 208 | ***80*** |  | 103 | ***29*** | 24 | ***9*** |  |  |
