# Supplementary material for: Prevention and control of mosquito-borne arboviral diseases: lessons learned from a school-based intervention in Brazil (Zikamob)
Source: BMC Public Health. 2022 Feb 8;22:255. doi: 10.1186/s12889-022-12554-w (PMC8822808; doi:10.1186/s12889-022-12554-w)
Supplement: Supplementary file 2 — Additional file 2. [file 12889_2022_12554_MOESM2_ESM.docx]

Supplementary Table 2 - Results of school-based intervention for arboviruses, performed in the city of Campina Grande, Paraíba, Brazil. Frequency and percentage for each dependent variable in the unpaired sample (Study B). Pearson's chi-square test was performed comparing students (ST) and teachers (TCH), independently, before and after the intervention. The analysis was also made by comparing the frequencies in the group of students and teachers (ST x TCH). Abbreviations: N - absolute population number; n - number in the sample; % - percentage of valid responses excluding missing data.

| **DEPENDENT VARIABLES** | | **STUDY B - UNPAIRED ANALYSIS (N=626)** | | | | | | | | | | |
| --- | --- | --- | --- | --- | --- | --- | --- | --- | --- | --- | --- | --- |
|  |  | **Students (ST)** | | | | | **Teachers (TCH)** | | | | | **ST X TCH** |
|  |  | **Pre** | | **Post** | | **p** | **Pre** | | **Post** | | **p** | **p** |
|  |  | **n** | ***%*** | **n** | ***%*** |  | **n** | ***%*** | **n** | ***%*** |  |  |
| D1 - Water reservoirs stay open | Yes | 11 | *5* | 30 | ***15*** | **0.001*** | 6 | ***2*** | 0 | ***0*** | 0.124 | 0.440 |
|  | No | 178 | *73* | 154 | ***75*** |  | 49 | ***20*** | 20 | ***10*** |  |  |
| D2 - Family survey reservoirs at least once a month | No | 64 | *19* | 33 | ***16*** | **0.021*** | 28 | ***8*** | 3 | ***1*** | 0.127 | 0.277 |
|  | Yes | 178 | *53* | 159 | ***75*** |  | 66 | ***20*** | 17 | ***8*** |  |  |
| D3 - Family cleans water tanks | No | 33 | *10* | 19 | ***9*** | 0.142 | 20 | ***6*** | 3 | ***1*** | 0.588 | **0.033*** |
|  | Yes | 192 | *60* | 173 | ***82*** |  | 74 | ***23*** | 16 | ***8*** |  |  |
| D4 -Open trash cans | Yes | 60 | *16* | 42 | ***16*** | 0.108 | 18 | ***5*** | 2 | ***1*** | 0.315 | 0.078 |
|  | No | 190 | *52* | 191 | ***74*** |  | 96 | ***26*** | 23 | ***9*** |  |  |
| D5 -Family separates solid waste for recycling | No | 158 | *44* | 96 | ***37*** | **<0.001*** | 56 | ***15*** | 12 | ***5*** | 0.938 | 0.492 |
|  | Yes | 91 | *25* | 138 | ***53*** |  | 58 | ***16*** | 12 | ***5*** |  |  |
| D6 - Family donates recyclables to waste pickers | No | 138 | *38* | 88 | ***34*** | **<0.001*** | 57 | ***16*** | 7 | ***3*** | 0.063 | 0.915 |
|  | Yes | 111 | *31* | 145 | ***56*** |  | 57 | ***16*** | 17 | ***7*** |  |  |
| D7-Family inspects potted plants | No | 49 | *20* | 31 | ***16*** | **0.049*** | 10 | ***4*** | 3 | ***2*** | 0.724 | 0.198 |
|  | Yes | 136 | *54* | 143 | ***75*** |  | 56 | ***22*** | 13 | ***7*** |  |  |
| D8-Windows with screens | No | 216 | *60* | 108 | ***42*** | **<0.001*** | 91 | ***25*** | 17 | ***7*** | 0.143 | **0.008*** |
|  | Yes | 34 | *9* | 126 | ***49*** |  | 21 | ***6*** | 8 | ***3*** |  |  |
| D9- Family usually close windows at dawn and dusk | No | 75 | *21* | 17 | ***7*** | **<0.001*** | 47 | ***13*** | 5 | ***2*** | **0.044*** | **<0.001*** |
|  | Yes | 174 | *48* | 219 | ***84*** |  | 66 | ***18*** | 20 | ***8*** |  |  |
| D10-Capped Drains | No | 42 | *12* | 29 | ***11*** | 0.165 | 44 | ***12*** | 8 | ***3*** | 0.497 | **<0.001*** |
|  | Yes | 208 | *57* | 206 | ***79*** |  | 68 | ***19*** | 17 | ***7*** |  |  |
| D11- Family usually cleans vacant lots | No | 115 | *48* | 80 | ***40*** | **<0.001*** | 66 | ***28*** | 14 | ***7*** | 0.252 | **0.001*** |
|  | Yes | 39 | *16* | 100 | ***50*** |  | 18 | ***8*** | 7 | ***3*** |  |  |
| DC0- Breeding site - have found mosquito larvae in your home | Yes | 124 | *34* | 104 | ***40*** | 0.239 | 42 | ***12*** | 12 | ***5*** | 0.300 | 0.088 |
|  | No | 126 | *35* | 131 | ***50*** |  | 72 | ***20*** | 13 | ***5*** |  |  |
| DC1- When finding the breeding site- made or would dispose of water on land and in sunny location | No | 46 | *13* | 43 | ***17*** | 0.947 | 26 | ***7*** | 7 | ***3*** | 0.537 | 0.155 |
|  | Yes | 201 | *56* | 185 | ***73*** |  | 86 | ***24*** | 17 | ***7*** |  |  |
| DC2- When finding the breeding site - did or would sanitize with bleach and bushing | No | 18 | *5* | 16 | ***6*** | 0.940 | 5 | ***1*** | 0 | ***0*** | 0.286 | 0.129 |
|  | Yes | 230 | *64* | 210 | ***84*** |  | 109 | ***30*** | 25 | ***10*** |  |  |
| DC3- When finding the breeding site - did or would do inspection of the house | No | 10 | *3* | 14 | ***5*** | 0.341 | 5 | ***1*** | 2 | ***1*** | 0.454 | 0.974 |
|  | Yes | 237 | *66* | 222 | ***85*** |  | 109 | ***30*** | 23 | ***9*** |  |  |
| DC4- Finding the breeding site - warned or would warn neighbors | No | 11 | *3* | 16 | ***6*** | 0.260 | 2 | ***1*** | 0 | ***0*** | 0.503 | **0.040*** |
|  | Yes | 234 | *65* | 217 | ***84*** |  | 111 | ***31*** | 25 | ***10*** |  |  |
| DC5- Upon finding the breeding site, notified or would notify the Environmental Surveillance service | No | 149 | *42* | 74 | ***29*** | **<0.001*** | 47 | ***13*** | 5 | ***2*** | **0.038*** | 0.092 |
|  | Yes | 98 | *27* | 160 | ***62*** |  | 64 | ***18*** | 20 | ***8*** |  |  |
